# Aggregate index of systemic inflammation (AISI) in admission as a reliable predictor of mortality in COPD patients with COVID-19

**DOI:** 10.1186/s12890-023-02397-5

**Published:** 2023-03-31

**Authors:** Saeed Hosseninia, Hassan Ghobadi, Kara Garjani, Seyed Amir Hossein Hosseini, Mohammad Reza Aslani

**Affiliations:** 1 Lung Diseases Research Center, Ardabil University of Medical Sciences, Ardabil, 5615780011 Iran; 2 Faculty of Medicine, Ardabil University of Medical Sciences, Ardabil, Iran; 3 Applied Biomedical Research Center, Mashhad University of Medical Sciences, Mashhad, Iran

**Keywords:** Coronavirus, COVID-19, COPD, Aggregate index of systemic inflammation

## Abstract

**Background:**

The role of leukocytes and systemic inflammation indicators in predicting the severity and mortality of inflammatory diseases has been well reported, such as the neutrophil to lymphocyte ratio (NLR), platelet to lymphocyte ratio (PLR), monocyte to lymphocyte ratio (MLR), neutrophil/lymphocyte*platelet ratio (NLPR), derived neutrophil/lymphocyte ratio (dNLR), aggregate index of systemic inflammation (AISI), as well as systemic inflammation response index (SIRI) and systemic inflammation index (SII). The purpose of the present study was to investigate the prognostic role of systemic inflammatory indicators in the mortality of chronic obstructive pulmonary disease (COPD) patients with COVID-19.

**Methods:**

This retrospective study included 169 COPD patients hospitalized with COVID-19. Demographic, clinical, and laboratory data were obtained from the patients’ electronic records. The ability of systemic inflammation indeces to distinguish the severity of COVID-19 was determined by receiver operating characteristic (ROC) analysis, and survival probability was determined by the mean of Kaplan–Meier curves, with the endpoint being death.

**Results:**

ROC curves showed that the AUD level was significant for WBC, MLR, SIRI, and AISI. Interestingly, Kaplan-Meier survival curves revealed that survival was lower with higher MLR (HR = 2.022, 95% CI = 1.030 to 3.968, P < 0.05) and AISI (HR = 2.010, 95% CI = 1.048 to 3.855, P < 0.05) values. However, the multivariate Cox regression model showed that only AISI was significantly associated with survival.

**Conclusion:**

AISI in COPD patients with COVID-19 was a reliable predictor of mortality.

## Introduction

Although the clinical and epidemiological characteristics of coronavirus 19 disease (COVID-19) are well established, more studies are needed to understand the spectrum of SARS-CoV-2 infection. Immune system disorders and exacerbated inflammatory responses are two prominent features of COVID-19, especially in severe disease and critical illness [1]. Despite the difference in the severity of diseases, those with severe pneumonia and respiratory difficulties usually require intensive care unit hospitalization [2]. Acute respiratory distress syndrome (ARDS) is a condition that very severe patients may experience, resulting from a range of factors including intrapulmonary vascular events, severe lung endothelial injury, and intussusceptive neoangiogenesis [3, 4]. It has been observed that various chemokines, including monocyte chemoattractant protein-1 (MCP-1), that are secreted by cells such as monocytes, macrophages, epithelial, endothelial, and smooth muscle cells, are fundamental in inflammation, coagulation, and angiogenesis for those suffering from COVID-19 [5].

The use of different indicators to predict diseases severity and mortality is of great interest in treatment systems for effective treatment. Leukocyte and systemic inflammation indices are common and cost-effective indices with diagnostic and prognostic applications, such as neutrophil-to-lymphocyte ratio (NLR), platelet-to-lymphocyte ratio (PLR), monocyte-to-lymphocyte ratio (MLR), neutrophil/lymphocyte*platelet ratio (NLPR), derived neutrophil/lymphocyte ratio (dNLR), aggregate index of systemic inflammation (AISI), systemic inflammation response index (SIRI), and systemic inflammation index (SII) [6–8]. The predictive effect of some of these indicators in patients with COVID-19 has been well documented, such as NLR, SIRI, and leukocytes [8]. As comorbidities (such as cardiovascular, renal, liver, and respiratory disorders) play an important role in the severity of COVID-19 [9], it is important to investigate the role of systemic inflammation indicators in COVID − 19 patients with various comorbidities.

Chronic obstructive pulmonary disease (COPD) is an inflammatory respiratory disease characterized by airway obstruction [10]. Increased levels of inflammatory markers have been demonstrated in patients with COPD and asthma, such as interleukin (IL)-6, tumor necrosis factor-alpha (TNF-α), IL-1β, C-reactive protein (CRP) [11, 12], as well as adipocytokines such as visfatin, apelin, adipolin, and FABP4 [13, 14]. Since in patients with COVID-19, cytokine storm have been reported as a result of a maladjusted immune response, which can cause severe damage to the lung parenchyma, it seems that the coexistence of COPD with COVID-19 affects the intensified immune response. To our knowledge, no study has investigated the predictors of systemic inflammation indices in COPD patients with COVID-19. Therefore, in the current study, we sought to examine the role of leukocytes and systemic inflammation indicators in predicting disease severity and mortality in COPD patients with COVID-19.

## Method and data collection

This retrospective study included a total of 169 individuals who had COPD and were hospitalized for COVID-19 during the period from August to December 2020. The diagnosis of COVID-19 was based on the PCR test. Two trained medical students collected demographic, clinical, and laboratory data such as age, sex, comorbidities, medical history, duration of hospitalization, disease outcome (recovery or death), and laboratory tests recorded in an electronic medical system, including total white blood count (WBC) and differential cells, hemoglobin (Hb), hematocrit (Hct), platelet (Plt), ferritin, erythrocyte sedimentation rate (ESR), urea, creatinine (Cr), sodium (Na), potassium (K), prothrombin time (PT), partial thromboplastin time (PTT), international normalized ratio (INR), aspartate transaminase (AST), lactate dehydrogenase (LDH), blood glucose (BG), alanine transaminase (ALT), D-dimer, and alkaline phosphatase (ALP). According to the World Health Organization’s (WHO) directives, the severity of COVID-19 was categorized into three levels: Moderate, for those in non-ICU with severe pneumonia necessitating oxygen therapy; Severe, for those in ICU with mild ARDS; and Very Severe, for those in ICU with severe ARDS [15].

Also, systemic inflammation indices were calculated for all subjects, such as NLR, PLR, MLR, dNLR (neutrophils/(white blood cells - neutrophils)), NLPR (neutrophil/(lymphocyte * platelet)), SIR-I ((neutrophils*monocytes)/lymphocytes), SII ((neutrophils * platelets)/lymphocytes), and AISI ((neutrophils*monocytes* platelet)/lymphocytes).

### Data analysis

The results are reported as mean ± standard deviation (SD) or median and interquartile range (IQR) (data with normal or abnormal distribution, respectively). Independent t-tests (normal distribution) or Mann-Whitney tests (non-normal distribution) were used to compare the data. To calculate the cutoff, sensitivity, or specificity of the values based on the Youden index, receiver operating characteristic (ROC) curve tests were performed. Univariate analysis of the Charlson index was used to determine the total WBC count to prevent linear bias. Therefore, WBC and differential cells were reported to have adjusted values according to Charlson’s index. Survival analysis was performed for all patients, and the hospitalization time was defined as time zero. Survival probability was estimated based on systemic inflammation indices with the average of Kaplan–Meier curves, and death was described as the endpoint. Finally, the Cox proportional hazards regression test was performed. Statistical significance was set at P < 0.05. SPSS software version 21 and MedCalc version 19.4.1 were used for data analysis.

## Results

### Demographics characteristics and clinical outcomes

The data of 169 COPD patients hospitalized with COVID-19 are summarized in Table 1. The mean age of the patients was 69.27 ± 12.90, and the length of hospitalization was 8.49 ± 6.97. Most of the admitted patients were male (58%). The results based on disease severity were as follows: moderate (63.3%), severe (11.2%), and very severe (25.5%). In addition, the comorbidities reported by the patients were cardiovascular (30.8%), renal (7.7%), diabetic (29.6%), cancer (3%), and liver (1.8%). Of 169 COPD patients in the study, 123 (72.8%) recovered and 46 (27.8%) died.

**Table 1:** Demographic, hematological, and blood cell count-derived inflammation indexes of COVID-19

variables	Normal range	COPD patients with COVID-19 (n = 169)
Age	-	69.27 ± 12.90
Sex	-	
Male, N (%) Female, N (%)		98 (58) 71 (42)
Hospitalization stay	-	8.49 ± 6.97
WBC (×10^9^/L)	3.5–9.5	7.40 (4.90–10.80)
Adjusted WBC (×10^9^/L)		8.45 ± 0.07
Adjusted Neutrophil (×10^9^/L)	1.8–6.3	6.62 ± 0.91
Adjusted Lymphocyte (×10^9^/L)	1.1–3.2	1.41 ± 0.82
Adjusted Monocyte (×10^9^/L)	0.2–0.3	0.27 ± 0.17
Hb (mg/ml)	11.5–15	13.27 ± 2.46
Hct (%)	36–48	40.53 ± 7.12
PLT (×10^9^/L)	125–350	189 (145–250)
Adjusted Plt	125–350	202.28 ± 11.89
PT (s)	11-13.5	13.68 ± 3.54
PTT	30–40	35.15 ± 11.04
INR	0.8–1.1	1.18 ± 0.50
ALT (IU/L)	7–40	32 (22.25-51)
AST (IU/L)	0–45	52 (34–73)
LDH (IU/L)	114–240	640 (470–789)
Ferritin (µg/L)	11–330	494 (138–937)
ESR (mm/hr)	0–29	45 (25–58)
BS (mg/ml)	70–100	120 (102–163)
Urea (mg/mL)	6–24	45 (32.5–69.5)
Cr (mg/mL)	0.5–1.2	1.1 (0.90–1.50)
D.Dimer (mg/L)	0-0.5	0.64 (0.25–0.90)
ALP (IU/L)	44–147	183 (144–237)
Na (mEq/L)	135–145	140 (137–142)
K (mEq/L)	3.5–5.3	4.00 (3.80–4.60)
NLR		7.03 ± 5.52
PLR		201.08 ± 137.09
MLR		0.25 ± 0.22
dNLR		4.93 ± 3.20
NLPR		0.03 ± 0.02
SIRI		1.72 ± 1.66
SII		1419 ± 1112
AISI		349.00 ± 340.62
Severity	-	
Moderate, N (%) Severe, N (%) Very severe, N (%)		107 (63.3) 19 (11.2) 43 (25.4)
Comorbidities	-	
Cardiovascular Disease (%) Kidney Disease (%) Diabetes (%) Cancer (%) Liver (%)		30.8 7.7 29.6 3.0 1.8
Charlson Comorbidity index		4.30 ± 1.77
Outcome Survival, N (%) Death, N (%)	-	123 (72.8) 46 (27.2)

AISI: aggregate index of systemic inflammation, ALP: Alkaline phosphatase, ALT: alanine transaminase, AST: aspartate transaminase, BS: blood sugar, Cr: creatinine, dNLR: derived neutrophil/lymphocyte ratio, ESR: erythrocyte sedimentation rate, Hb: hemoglobin, Hct: hematocrit, INR: international normalized ratio, LDH: lactate dehydrogenase, MLR: monocyte to lymphocyte ratio, NLPR: neutrophil/lymphocyte*platelet ratio, NLR: neutrophil to lymphocyte ratio, Plt: platelet, PLR: platelet to lymphocyte ratio, PT: Prothrombin time, PTT: Partial thromboplastin time, SIR-I: systemic inflammation response index, SII: systemic inflammation index, WBC: white blood cell

Increased levels of AST, LDH, ferritin, ESR, BS, urea, D-dimer, and ALP were evident in COPD patients with COVID-19 at admission, whereas the levels of other laboratory tests were within the normal range, such as WBC, Hb, Hct, PT, PTT, Plt, INR, Na, K, and ALT (Table 1).

The mean systemic inflammation indices observed in COPD patients were NLR (7.03 ± 5.52), PLR (201.08 ± 137.09), MLR (0.25 ± 0.22), dNLR (4.93 ± 3.20), NLPR (0.03 ± 0.02), SIRI (1.72 ± 1.66), SII (1419 ± 1112), and AISI (349.00 ± 340.62).

### Laboratory parameters based on outcome

It was found that the mean age (P < 0.05), length of hospitalization (P < 0.01), Hct (P < 0.05), LDH (P < 0.01), urea (P < 0.001), MLR (P < 0.05), SIRI (P < 0.05), and AISI (P < 0.05) in COPD patients who died were significantly higher than those who recovered. Instead, the mean WBC (P < 0.05) and Plt (P < 0.05) in the dead patients were significantly lower than those in the recovered subjects (Table 2).

**Table 2:** Demographic, hematological, and blood cell count-derived inflammation indexes of COVID-19 in survivor and non-survivor patients. Abbreviations similar to Table 1.

variables	COPD patients with COVID-19		P-value
	Survival (N = 274)	Death (N = 26)	
Age	68.06 ± 12.93	72.50 ± 12.37	0.046
Sex			0.727
Male, N (%) Female, N (%)	70 (56.9) 53 (43.1)	28 (60.9) 18 (39.1)	
Hospitalization stay	7.83 ± 5.14	11.89 ± 9.86	0.001
WBC (×10^9^/L)	8.47 ± 0.08	8.42 ± 0.06	0.000
Neutrophil (×10^9^/L)	6.60 ± 0.89	6.66 ± 0.96	0.716
Lymphocyte (×10^9^/L)	1.46 ± 0.81	1.30 ± 0.86	0.268
Monocyte (×10^9^/L)	0.26 ± 0.16	0.30 ± 0.18	0.139
Hb (mg/ml)	13.10 ± 2.35	13.71 ± 2.71	0.149
Hct (%)	39.68 ± 6.17	42.78 ± 8.89	0.034
PLT (×10^9^/L)	203.70 ± 12.15	198.50 ± 10.35	0.011
PT (s)	13.52 ± 3.85	14.10 ± 2.51	0.366
PTT	33.91 ± 8.20	38.41 ± 15.99	0.084
INR	1.15 ± 0.54	1.25 ± 0.37	0.246
ALT (IU/L)	55.61 ± 76.99	49.41 ± 64.60	0.644
AST (IU/L)	70.74 ± 82.38	91.28 ± 134.17	0.257
LDH (IU/L)	143.18 ± 264.79	827.74 ± 461.99	0.003
Ferritin (µg/L)	627.71 ± 561.86	548.45 ± 589.16	0.520
ESR (mm/hr)	44.28 ± 22.83	39.89 ± 28.28	0.402
BG (mg/ml)	140.79 ± 68.54	157.00 ± 68.92	0.201
Urea (mg/mL)	48.96 ± 25.73	67.08 ± 33.69	0.000
Cr (mg/mL)	1.39 ± 1.24	1.53 ± 0.77	0.488
D.Dimer (mg/L)	719.95 ± 699.79	797.00 ± 670.12	0.753
ALP (IU/L)	202.29 ± 112.65	226.09 ± 148.85	0.324
Na (mEq/L)	139.50 ± 3.81	139.44 ± 4.73	0.938
K (mEq/L)	4.12 ± 0.53	4.32 ± 0.94	0.174
NLR	6.68 ± 4.99	7.98 ± 6.72	0.174
PLR	192.78 ± 123.12	223.27 ± 168.27	0.199
MLR	0.22 ± 0.19	0.31 ± 0.27	0.022
dNLR	4.86 ± 3.23	5.10 ± 3.13	0.668
NLPR	0.03 ± 0.02	0.04 ± 0.03	0.130
SIRI	1.55 ± 1.44	2.19 ± 2.08	0.025
SII	1357 ± 1007	1587 ± 1353	0.231
AISI	316.30 ± 298.96	436.45 ± 424.37	0.041
Severity			0.000
Moderate, N (%) Severe, N (%) Very severe, N (%)	102 (95.3) 17 (89.5) 4 (9.3)	5 (4.7) 2 (10.5) 39 (90.7)	
Comorbidities			
Cardiovascular Disease (%) Kidney Disease (%) Diabetes (%) Cancer (%) Liver (%)	29.3 5.7 27.6 2.4 2.4	34.8 13 34.8 4.3 0	0.304 0.105 0.449 0.614 0.563

### Receiver operating characteristics (ROC)

**Fig. 1:**
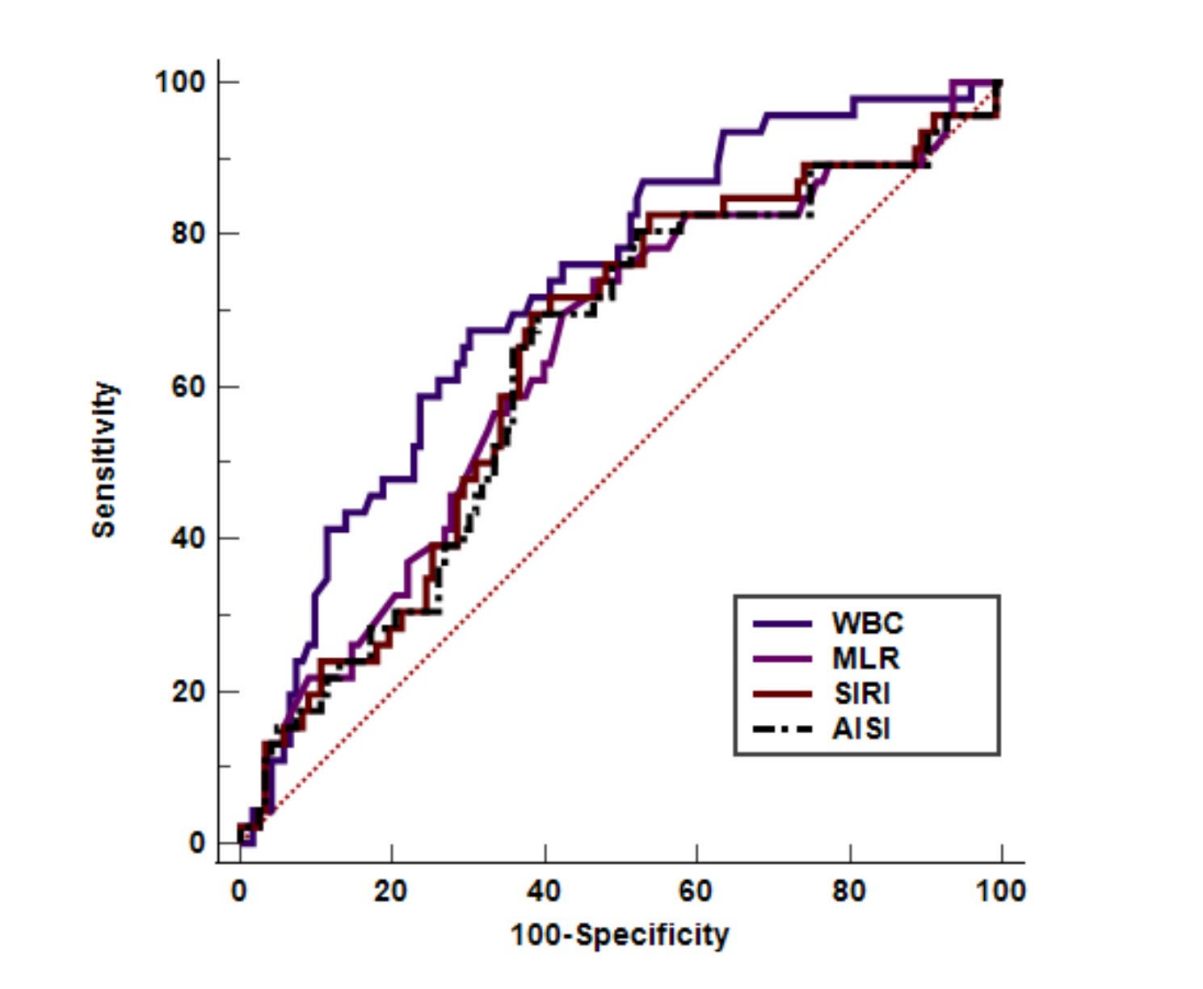
Receiver operating characteristics curve of COPD patients with COVID-19 for WBC, MLR, AISI, and SIRI. AISI: aggregate index of systemic inflammation, MLR: monocyte/lymphocyte ratio, SII: systemic inflammation index, SIRI: systemic inflammation response index, WBC: white blood cell

**Fig. 2:**
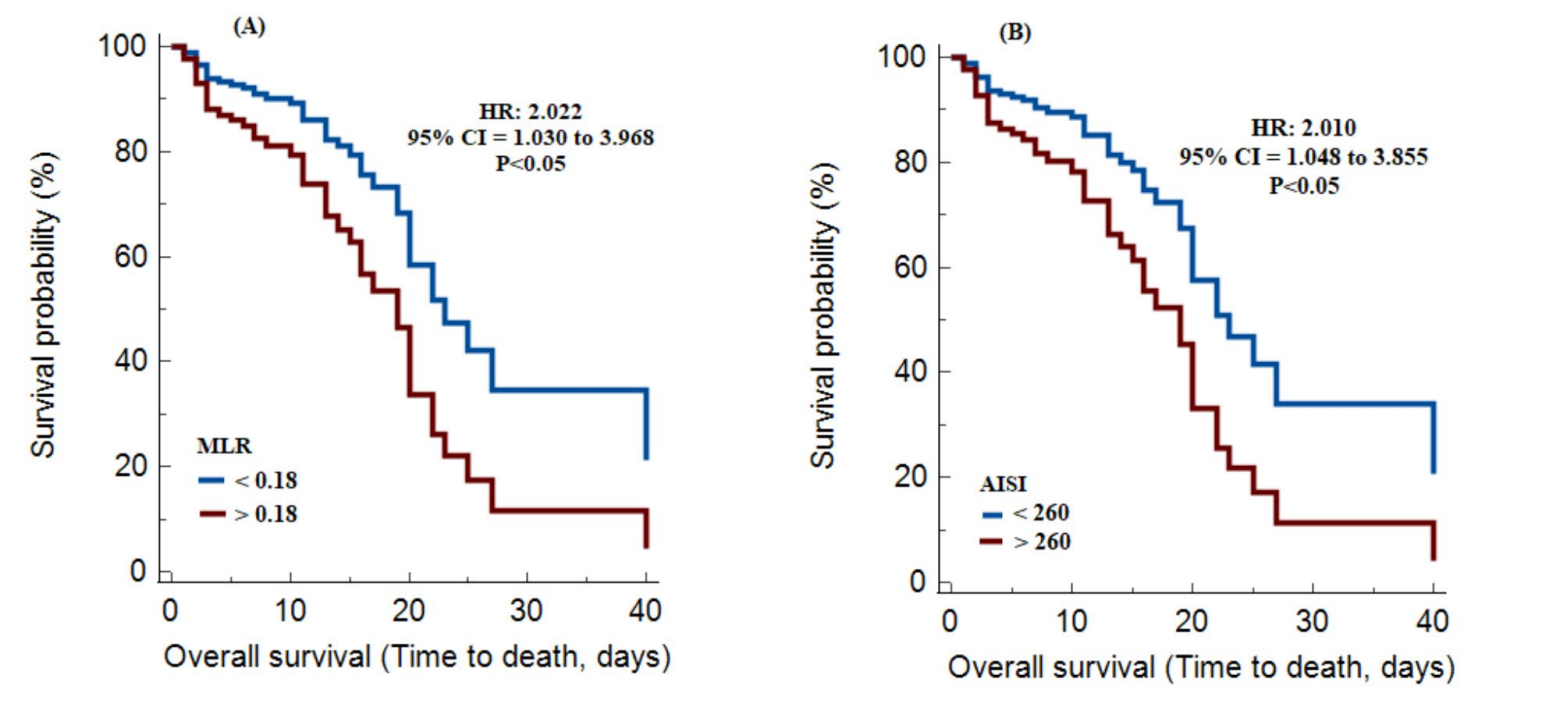
Kaplan–Meier survival curves during hospitalization of COPD patients with different cut-off values of the systemic inflammation indexes investigated. (**A**): MLR and (**B**): AISI. AISI: aggregate index of systemic inflammation, MLR: monocyte/lymphocyte ratio

In COPD patients hospitalized with COVID-19, the appropriate cut-off values for leukocyte and systemic inflammatory indices based on the ROC curve were as follows: WBC count (≤ 8.43), neutrophil count (> 6.56), lymphocyte count (≤ 1.51), monocyte count (> 0.24), NLR (> 4.44), PLR (> 123.51), MLR (> 0.18), dNLR (> 3.34), NLPR (> 0.02), SIRI (> 1.29), SII (> 939), and AISI (> 260) (Table 3). However, the AUD was significant for WBC (0.725), MLR (0.639), SIRI (0.640), and AISI (0.630) (Fig. 1). Interestingly, the AUD was significantly higher in the SIRI than in the AISI (P < 0.05). The Kaplan-Meier survival curves revealed that survival was significantly lower with high levels of MLR (HR = 2.022, 95% CI = 1.030 to 3.968, P < 0.05) and AISI (HR = 2.010, 95% CI = 1.048 to 3.855, P < 0.05) (Fig. 2). In contrast, multivariate Cox regression models showed that only AISI (HR = 2.010, 95% CI = 1.048 to 3.855, P < 0.05) was significantly associated with survival.

**Table 3:** Receiver operating characteristics (ROC) curves and prognostic accuracy of blood cell count-derived inflammation indexes in COVID-19

variables	AUC	95% CI	p-Value	Cut-off	Sensitivity	Specificity (%)
WBC adjusted	0.725	0.652 to 0.791	0.000	≤ 8.43	67.4	69.9
Neu adjusted	0.529	0.451 to 0.607	0.544	> 6.56	73.9	41.5
Lym adjusted	0.575	0.497 to 0.651	0.119	≤ 1.51	80.4	44.7
Mono adjusted	0.553	0.474 to 0.629	0.277	> 0.24	71.7	44.7
NLR	0.563	0.485 to 0.639	0.192	> 4.44	78.3	43.1
PLR	0.555	0.477 to 0.632	0.252	> 123.51	84.8	38.2
MLR	0.639	0.562 to 0.711	0.004	> 0.18	73.9	53.7
dNLR	0.543	0.464 to 0.619	0.377	> 3.34	76.1	39.8
NLPR	0.573	0.495 to 0.649	0.131	> 0.02	78.3	43.1
SIRI	0.640	0.563 to 0.712	0.004	> 1.29	69.6	61.8
SII	0.550	0.472 to 0.627	0.299	> 939	73.9	46.3
AISI	0.630	0.552 to 0.703	0.007	> 260	69.6	61.0

AISI: aggregate index of systemic inflammation, dNLR: derived neutrophil/lymphocyte ratio, Lym: lymphocyte, Mono: monocyte, MLR: monocyte/lymphocyte ratio, Neu: neutrophil, NLPR: neutrophil/lymphocyte*platelet ratio, NLR: neutrophil/lymphocyte ratio, PLR: platelet/lymphocyte ratio, SII: systemic inflammation index, SIRI: systemic inflammation response index, WBC: white blood cell

## Discussion

The current study showed that in deceased COPD patients with COVID-19, significantly increased mean length of hospitalization, Hct, LDH, urea, MLR, SIRI, and AISI were evident compared to those in recovered patients. In addition, based on the ROC curves, the AUD was significant for WBC, MLR, SIRI, and AISI. Kaplan-Meier survival curve results revealed that survival was lower with higher MLR and AISI values. But the multivariate Cox regression model showed that AISI was only significantly associated with survival.

Nowadays, although our knowledge of the epidemiological and clinical characteristics of patients with COVID-19 has increased, information related to the clinical spectrum of COVID-19 is limited. Middle-aged and elderly patients have a poor prognosis of COVID-19 due to comorbidities such as cardiovascular disease, hypertension, diabetes, and respiratory disorders [16]. The risk factors associated with the prognosis of various diseases, such as COVID-19, must be considered for effective treatment planning. Some studies have used leukocyte count and systemic inflammation indices to predict mortality in COVID-19 patients [8]. To the best of our knowledge, reports of all systemic inflammatory indicators for predicting mortality in COPD patients with COVID-19 have not been done. Our results showed that WBC count and some indeces of systemic inflammation at admission were associated with mortality risk in COPD patients with COVID-19.

Much evidence have shown that cytokine storm syndrome may occur in patients with COVID-19, especially in those with severe disease. Although the immune response is critical for controlling SARS-CoV-2 infection, maladaptive immune responses may be associated with increased mortality [17, 18]. The results showed that in deceased COPD patients, old age and long duration of hospitalization, as well as increased values of LDH, urea, MLR, SIRI, and AISI were evident. COPD patients are mostly older and have comorbidities [19]. The results of the current study also revealed that comorbidities such as cardiovascular disease, diabetes, and hypertension were present in COPD patients with COVID-19. Aging and some comorbidities, such as diabetes, are low-grade inflammatory states with increased levels of proinflammatory markers (IL-6 and TNF-α) [20–22]. On the other hand, COPD is considered a systemic inflammation with increased levels of inflammatory markers such as CRP, IL-6, and TNF-α [23]. Overall, it seems that in COPD patients with COVID-19, the inflammatory response affects disease severity, although the results are contradictory [24].

In most studies, white blood cell counts in patients with COVID-19 have been reported to be within the normal range, although high leukocyte levels have been associated with high mortality [16]. Increased levels of neutrophils and decreased levels of lymphocytes have been observed in patients with severe disease. The results of the current study are, at least in part, consistent with the above results. Leukocyte and systemic inflammation indices such as NLR, PLR, MLR, dNLR, NLPR, SIRI, SII, and AISI have been used in previous studies to predict the mortality of COVID-19 patients [8]. Studies have shown that most inflammatory indices are associated with mortality in COVID-19 patients [7]. However, the current results demonstrated that WBC count, MLR, SIRI, and AISI were significantly associated with mortality in COPD patients with COVID-19. Multivariate Cox regression analysis revealed that only the AISI remained significantly associated with survival. In patients with severe COVID-19 pneumonia, the results reflected that neutrophilia and lymphopenia were more evident. In addition, the NLR in the early stages of SARS-CoV-2 infection has been a reliable predictor of patient mortality, although a decrease in granulocytes has been reported in some studies [25]. The current study showed that in COVID-19 patients with different comorbidities, systemic inflammatory indicators predicting mortality might change. The AISI was a reliable predictor of mortality in COPD patients with COVID-19. Perhaps the difference in the results was due to the pathophysiology of COPD or the interaction between COVID-19 and COPD, which requires further invastigation. In SARS-COv2 infection, early suppression of the immune system occurs, such as lymphopenia and suppression of interferons. Under these conditions, there is a possibility of a more aggressive immune response with increased levels of inflammatory markers such as CRP, IL-6, TNF-alpha, D-dimer, IL-1β, IL-2, and IL-17 [26, 27]. Immune system hyperactivity with involvement of the lung parenchyma may lead to acute respiratory distress syndrome in patients with severe COVID-19. This condition is partially similar to the macrophage response that develops in COPD patients [19]. Perhaps the interaction between COVID-19 and COPD has a significant role in the type of systemic inflammatory response, which requires further studies.

The current study had some limitations as follows:1) it was a single-center study, 2) different variants of SARS-CoV-2 may have influenced the results of the study, 3) each patient was hospitalized with different severities of COVID-19, and 4) data were collected from the electronic registration system.

In conclusion, the results showed that systemic inflammation indices in COPD patients with COVID-19 were reliable predictors of disease severity and mortality. Unlike other studies that reported NLR for predicting mortality in patients with COVID-19, in COPD patients with COVID-19, AISI was a reliable indicator of mortality. In COVID-19 patients, prognostic systemic inflammatory indices should be carefully considered based on comorbidities.

## Data Availability

The data sets used and/or analyzed during the current study are available from the corresponding author on reasonable request.
